# Poly[hexa­aqua­bis(μ_3_-terephthalato)(μ_2_-terephthalato)diytterbium(III)]

**DOI:** 10.1107/S160053680905171X

**Published:** 2009-12-09

**Authors:** Sun Feng

**Affiliations:** a School of Chemistry and Environment, South China Normal University, Guangzhou 510631, People’s Republic of China

## Abstract

In the title two-dimensional coordination polymer, [Yb_2_(C_8_H_4_O_4_)_3_(H_2_O)_6_]_*n*_, the unique Yb^III^ ion is eight-coordinated in a distorted dodeca­hedral coordination geometry by three water O atoms and five O atoms from carboxyl­ate groups belonging to four different terephthalate ligands. One of the terephthalate ligands is located around an inversion center. The coordination polymers are parallel to (121) and are connected by O—H⋯O hydrogen bonds into a three-dimensional framework.

## Related literature

For the isostructural erbium(III) and lutetium(III) complexes, see: Daiguebonne *et al.* (2006 ▶); Xie *et al.* (2008 ▶).
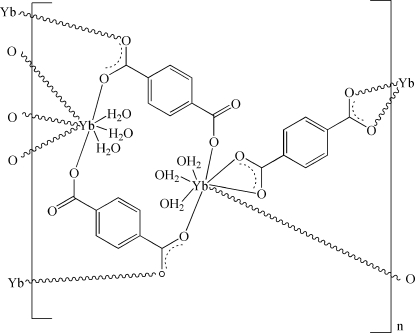


## Experimental

### 

#### Crystal data

[Yb_2_(C_8_H_4_O_4_)_3_(H_2_O)_6_]
*M* *_r_* = 473.26
Triclinic, 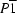

*a* = 7.8413 (7) Å
*b* = 9.5545 (8) Å
*c* = 10.6561 (9) Å
α = 68.827 (1)°
β = 71.024 (1)°
γ = 75.206 (1)°
*V* = 695.34 (10) Å^3^
*Z* = 2
Mo *K*α radiation
μ = 6.77 mm^−1^
*T* = 296 K
0.21 × 0.18 × 0.17 mm

#### Data collection

Bruker APEXII area-detector diffractometer
Absorption correction: multi-scan (*SADABS*; Sheldrick, 1996 ▶) *T*_min_ = 0.251, *T*_max_ = 0.316
3564 measured reflections
2444 independent reflections
2323 reflections with *I* > 2σ(*I*)
*R*_int_ = 0.015

#### Refinement

*R*[*F* ^2^ > 2σ(*F* ^2^)] = 0.020
*wR*(*F* ^2^) = 0.048
*S* = 1.04
2444 reflections
217 parameters
9 restraints
H atoms treated by a mixture of independent and constrained refinement
Δρ_max_ = 0.82 e Å^−3^
Δρ_min_ = −0.73 e Å^−3^

### 

Data collection: *APEX2* (Bruker, 2004 ▶); cell refinement: *SAINT* (Bruker, 2004 ▶); data reduction: *SAINT*; program(s) used to solve structure: *SHELXS97* (Sheldrick, 2008 ▶); program(s) used to refine structure: *SHELXL97* (Sheldrick, 2008 ▶); molecular graphics: *SHELXTL* (Sheldrick, 2008 ▶); software used to prepare material for publication: *SHELXL97*.

## Supplementary Material

Crystal structure: contains datablocks I. DOI: 10.1107/S160053680905171X/gk2241sup1.cif

Structure factors: contains datablocks I. DOI: 10.1107/S160053680905171X/gk2241Isup2.hkl

Additional supplementary materials:  crystallographic information; 3D view; checkCIF report

## Figures and Tables

**Table 1:** Hydrogen-bond geometry (Å, °)

*D*—H⋯*A*	*D*—H	H⋯*A*	*D*⋯*A*	*D*—H⋯*A*
O1*W*—H1*W*⋯O2*W*^i^	0.81 (4)	2.35 (3)	3.123 (5)	158 (6)
O1*W*—H1*W*⋯O3	0.81 (4)	2.52 (4)	3.104 (5)	130 (4)
O1*W*—H2*W*⋯O5^ii^	0.82 (4)	1.91 (5)	2.714 (4)	170 (5)
O2*W*—H3*W*⋯O1^iii^	0.82 (5)	1.97 (5)	2.771 (4)	167 (5)
O2*W*—H4*W*⋯O1^iv^	0.82 (3)	2.17 (3)	2.900 (5)	149 (5)
O3*W*—H5*W*⋯O6^v^	0.82 (3)	2.08 (2)	2.846 (4)	156 (4)
O3*W*—H6*W*⋯O1^iv^	0.82 (4)	1.95 (4)	2.746 (4)	165 (5)

Symmetry codes: (i) 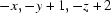; (ii) 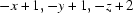; (iii) 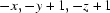; (iv) 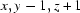; (v) 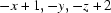.

**Fig. 1.**
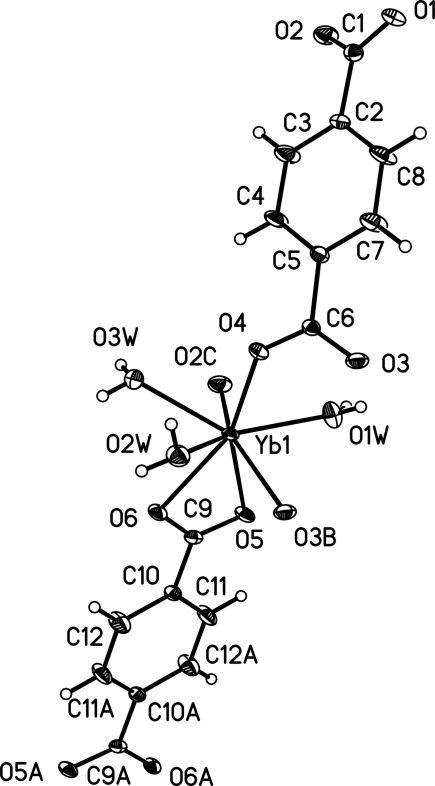
ORTEP view of the asymmetric unit of the title compound with displacement ellipsoids drawn at the 30% probability level. Symmetry codes: (A) 1 - x, -y, 3 - z.;(B) -x, 1 - y, 2 - z; (C) 1 - x, 1 - y, 1 - z.

**Fig. 2.**
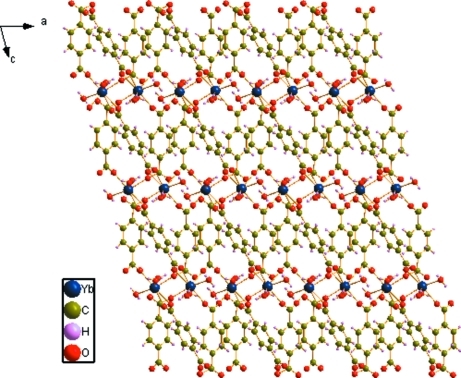
A view of the three-dimensional structure of the title compound. Hydrogen atoms are omitted for clarity.

## supplementary crystallographic information

### Comment

Terephthalic acid as a dicarboxylate ligand may exhibit various coordination modes with transition metals or lanthanide ions that result in one-, two- or three-dimensional metal-organic frameworks (MOFs). As an extension of this research we report here the structure of the title two-dimensional coordination polymer of Yb^III^ which is isostructural with Lu^III^ and Er^III^ complexes (Daiguebonne *et al.*, 2006; Xie *et al.*, 2008) .

The asymmetric unit of the title compound (Fig. 1) contains one Yb^III ^ion, one and a half of terephthalate ligand, and three coordinated water molecules. The Yb^III^ ion is eight-coordinate with a dodecahedral coordination polyhedron made of three oxygen atoms from three coordination water molecules and five oxygen atoms from carboxylate groups of four different terephthalate ligands.

The coordination polymers are joined by O—H···O hydrogen bonds between coordinated water molecules and carboxylate groups of terephthalate ligands (Table 1).

### Experimental

A mixture of AgNO_3_(0.057 g, 0.33 mmol), Yb_2_O_3_(0.116 g, 0.33 mmol), 2-pyrazinecarboxylic acid(0.165 g, 1.33 mmol), terephthalic acid (0.166 g, 1.0 mmol), H_2_O(7 ml), and HClO_4_(0.257 mmol)(pH 2) was sealed in a 20 ml Teflon-lined reaction vessel at 443 K for 6 days and then slowly cooled to room temperature. The product was collected by filtration, washed with water and air-dried. Colorless block crystals were suitable for X-ray analysis were obtained.

### Refinement

H atoms bonded to C atoms were positioned geometrically and refined as riding, with C—H = 0.93 Å and *U*_iso_(H) = 1.2 *U*_eq_(C). H atoms of the water molecules were found from difference Fourier maps and refined with restraints imposed on the O-H and H···H distances [O—H = 0.82 (1) Å and H···H = 1.29 (1) Å] and displacement parameters set to 1.5*U*_eq_(O).

### Crystal data

**Table:**

[Yb_2_(C_8_H_4_O_4_)_3_(H_2_O)_6_]	*Z* = 2
*M**_r_* = 473.26	*F*(000) = 452
Triclinic, *P*1	*D*_x_ = 2.260 Mg m^−^^3^
Hall symbol: -P 1	Mo *K*α radiation, λ = 0.71073 Å
*a* = 7.8413 (7) Å	Cell parameters from 2800 reflections
*b* = 9.5545 (8) Å	θ = 2.6–27.8°
*c* = 10.6561 (9) Å	µ = 6.77 mm^−^^1^
α = 68.827 (1)°	*T* = 296 K
β = 71.024 (1)°	Block, colorless
γ = 75.206 (1)°	0.21 × 0.18 × 0.17 mm
*V* = 695.34 (10) Å^3^	

### Data collection

**Table:**

Bruker APEXII area-detector diffractometer	2444 independent reflections
Radiation source: fine-focus sealed tube	2323 reflections with *I* > 2σ(*I*)
graphite	*R*_int_ = 0.015
Detector resolution: 0 pixels mm^-1^	θ_max_ = 25.2°, θ_min_ = 2.1°
φ and ω scan	*h* = −9→7
Absorption correction: multi-scan (*SADABS*; Sheldrick, 1996)	*k* = −10→11
*T*_min_ = 0.251, *T*_max_ = 0.316	*l* = −12→11
3564 measured reflections	

### Refinement

**Table:**

Refinement on *F*^2^	Primary atom site location: structure-invariant direct methods
Least-squares matrix: full	Secondary atom site location: difference Fourier map
*R*[*F*^2^ > 2σ(*F*^2^)] = 0.020	Hydrogen site location: inferred from neighbouring sites
*wR*(*F*^2^) = 0.048	H atoms treated by a mixture of independent and constrained refinement
*S* = 1.04	*w* = 1/[σ^2^(*F*_o_^2^) + (0.0237*P*)^2^ + 0.8814*P*] where *P* = (*F*_o_^2^ + 2*F*_c_^2^)/3
2444 reflections	(Δ/σ)_max_ = 0.003
217 parameters	Δρ_max_ = 0.82 e Å^−^^3^
9 restraints	Δρ_min_ = −0.73 e Å^−^^3^

### Special details

**Table:**

Geometry. All e.s.d.'s (except the e.s.d. in the dihedral angle between two l.s. planes) are estimated using the full covariance matrix. The cell e.s.d.'s are taken into account individually in the estimation of e.s.d.'s in distances, angles and torsion angles; correlations between e.s.d.'s in cell parameters are only used when they are defined by crystal symmetry. An approximate (isotropic) treatment of cell e.s.d.'s is used for estimating e.s.d.'s involving l.s. planes.
Refinement. Refinement of *F*^2^ against ALL reflections. The weighted *R*-factor *wR* and goodness of fit *S* are based on *F*^2^, conventional *R*-factors *R* are based on *F*, with *F* set to zero for negative *F*^2^. The threshold expression of *F*^2^ > σ(*F*^2^) is used only for calculating *R*-factors(gt) *etc*. and is not relevant to the choice of reflections for refinement. *R*-factors based on *F*^2^ are statistically about twice as large as those based on *F*, and *R*- factors based on ALL data will be even larger.

### Fractional atomic coordinates and isotropic or equivalent isotropic displacement parameters (Å^2^)

**Table:**

	*x*	*y*	*z*	*U*_iso_*/*U*_eq_	
Yb1	0.26626 (2)	0.294523 (19)	1.006579 (16)	0.01940 (7)	
C1	0.2794 (6)	0.7548 (5)	0.1682 (4)	0.0244 (9)	
C2	0.2113 (6)	0.6900 (5)	0.3233 (4)	0.0231 (9)	
C3	0.3017 (8)	0.5574 (6)	0.3942 (5)	0.0546 (17)	
H3	0.3999	0.5039	0.3444	0.066*	
C4	0.2506 (8)	0.5012 (6)	0.5381 (5)	0.0501 (15)	
H4	0.3117	0.4094	0.5839	0.060*	
C5	0.1094 (6)	0.5813 (5)	0.6133 (4)	0.0238 (9)	
C6	0.0601 (5)	0.5283 (5)	0.7702 (4)	0.0210 (8)	
C7	0.0173 (7)	0.7138 (6)	0.5423 (5)	0.0465 (14)	
H7	−0.0791	0.7690	0.5917	0.056*	
C8	0.0665 (7)	0.7660 (6)	0.3982 (5)	0.0435 (13)	
H8	0.0001	0.8540	0.3519	0.052*	
C9	0.3969 (5)	0.1538 (5)	1.2422 (4)	0.0221 (9)	
C10	0.4506 (6)	0.0733 (5)	1.3751 (4)	0.0243 (9)	
C11	0.5442 (8)	0.1424 (5)	1.4211 (5)	0.0406 (13)	
H11	0.5754	0.2380	1.3678	0.049*	
C12	0.4091 (8)	−0.0693 (6)	1.4544 (5)	0.0411 (13)	
H12	0.3486	−0.1171	1.4234	0.049*	
O1	0.1745 (4)	0.8546 (4)	0.1024 (3)	0.0324 (7)	
O2	0.4422 (4)	0.7076 (4)	0.1137 (3)	0.0361 (8)	
O3	−0.0475 (4)	0.6148 (4)	0.8340 (3)	0.0317 (7)	
O4	0.1368 (5)	0.3988 (3)	0.8290 (3)	0.0336 (7)	
O5	0.4165 (4)	0.2898 (3)	1.1772 (3)	0.0276 (7)	
O6	0.3249 (4)	0.0831 (3)	1.1957 (3)	0.0271 (7)	
O1W	0.3026 (5)	0.5482 (4)	0.9349 (4)	0.0387 (8)	
H1W	0.221 (4)	0.617 (4)	0.914 (6)	0.058*	
H2W	0.393 (4)	0.589 (5)	0.908 (6)	0.058*	
O2W	0.0025 (5)	0.1676 (4)	1.0747 (4)	0.0358 (8)	
H3W	−0.038 (7)	0.168 (5)	1.013 (4)	0.054*	
H4W	0.030 (8)	0.0778 (19)	1.115 (5)	0.054*	
O3W	0.3438 (5)	0.0930 (3)	0.9110 (3)	0.0329 (7)	
H5W	0.451 (2)	0.065 (5)	0.877 (5)	0.049*	
H6W	0.303 (5)	0.014 (3)	0.957 (5)	0.049*	

### Atomic displacement parameters (Å^2^)

**Table:**

	*U*^11^	*U*^22^	*U*^33^	*U*^12^	*U*^13^	*U*^23^
Yb1	0.02266 (11)	0.02072 (11)	0.01111 (10)	−0.00012 (7)	−0.00580 (7)	−0.00153 (7)
C1	0.027 (2)	0.026 (2)	0.021 (2)	−0.0095 (19)	−0.0056 (18)	−0.0052 (18)
C2	0.026 (2)	0.028 (2)	0.015 (2)	−0.0081 (18)	−0.0059 (16)	−0.0031 (17)
C3	0.064 (4)	0.049 (3)	0.019 (2)	0.029 (3)	−0.001 (2)	−0.006 (2)
C4	0.065 (4)	0.039 (3)	0.017 (2)	0.027 (3)	−0.008 (2)	−0.002 (2)
C5	0.026 (2)	0.023 (2)	0.018 (2)	−0.0040 (18)	−0.0057 (17)	−0.0023 (17)
C6	0.023 (2)	0.022 (2)	0.018 (2)	−0.0043 (17)	−0.0086 (17)	−0.0035 (17)
C7	0.036 (3)	0.053 (3)	0.019 (2)	0.024 (2)	0.002 (2)	−0.003 (2)
C8	0.034 (3)	0.050 (3)	0.018 (2)	0.019 (2)	−0.005 (2)	0.003 (2)
C9	0.021 (2)	0.027 (2)	0.015 (2)	−0.0004 (17)	−0.0046 (16)	−0.0045 (18)
C10	0.030 (2)	0.023 (2)	0.018 (2)	0.0013 (18)	−0.0117 (17)	−0.0023 (17)
C11	0.070 (4)	0.028 (3)	0.029 (3)	−0.019 (2)	−0.028 (2)	0.007 (2)
C12	0.066 (4)	0.036 (3)	0.033 (3)	−0.020 (3)	−0.034 (3)	0.003 (2)
O1	0.0370 (18)	0.0362 (18)	0.0199 (15)	−0.0057 (15)	−0.0127 (14)	0.0009 (14)
O2	0.0302 (18)	0.046 (2)	0.0226 (16)	−0.0030 (15)	0.0006 (13)	−0.0083 (15)
O3	0.0330 (17)	0.0383 (19)	0.0170 (15)	0.0030 (14)	−0.0030 (13)	−0.0094 (14)
O4	0.054 (2)	0.0251 (17)	0.0212 (16)	0.0009 (15)	−0.0202 (15)	−0.0014 (13)
O5	0.0352 (17)	0.0276 (17)	0.0182 (15)	−0.0074 (13)	−0.0135 (13)	0.0027 (13)
O6	0.0391 (18)	0.0250 (16)	0.0196 (15)	−0.0059 (13)	−0.0179 (13)	0.0003 (13)
O1W	0.0313 (18)	0.0267 (18)	0.052 (2)	−0.0055 (14)	−0.0105 (17)	−0.0049 (16)
O2W	0.0395 (19)	0.0379 (19)	0.0327 (19)	−0.0096 (16)	−0.0140 (15)	−0.0074 (15)
O3W	0.0388 (19)	0.0277 (17)	0.0296 (18)	−0.0009 (14)	−0.0077 (15)	−0.0096 (14)

### Geometric parameters (Å, °)

**Table:**

Yb1—O2^i^	2.227 (3)	C6—O4	1.261 (5)
Yb1—O4	2.231 (3)	C7—C8	1.384 (6)
Yb1—O3^ii^	2.233 (3)	C7—H7	0.9300
Yb1—O1W	2.327 (3)	C8—H8	0.9300
Yb1—O3W	2.352 (3)	C9—O5	1.255 (5)
Yb1—O6	2.354 (3)	C9—O6	1.280 (5)
Yb1—O2W	2.438 (3)	C9—C10	1.487 (5)
Yb1—O5	2.450 (3)	C10—C12	1.376 (6)
Yb1—C9	2.773 (4)	C10—C11	1.389 (6)
C1—O1	1.252 (5)	C11—C12^iii^	1.378 (6)
C1—O2	1.259 (5)	C11—H11	0.9300
C1—C2	1.502 (5)	C12—C11^iii^	1.378 (6)
C2—C8	1.364 (6)	C12—H12	0.9300
C2—C3	1.370 (6)	O2—Yb1^i^	2.227 (3)
C3—C4	1.385 (6)	O3—Yb1^ii^	2.233 (3)
C3—H3	0.9300	O1W—H1W	0.81 (4)
C4—C5	1.373 (6)	O1W—H2W	0.82 (4)
C4—H4	0.9300	O2W—H3W	0.82 (5)
C5—C7	1.376 (6)	O2W—H4W	0.82 (3)
C5—C6	1.507 (5)	O3W—H5W	0.82 (3)
C6—O3	1.238 (5)	O3W—H6W	0.82 (4)
			
O2^i^—Yb1—O4	98.80 (12)	C5—C4—C3	119.9 (4)
O2^i^—Yb1—O3^ii^	144.25 (12)	C5—C4—H4	120.1
O4—Yb1—O3^ii^	98.76 (12)	C3—C4—H4	120.1
O2^i^—Yb1—O1W	76.69 (12)	C4—C5—C7	118.6 (4)
O4—Yb1—O1W	77.10 (12)	C4—C5—C6	120.8 (4)
O3^ii^—Yb1—O1W	77.26 (12)	C7—C5—C6	120.5 (4)
O2^i^—Yb1—O3W	73.41 (12)	O3—C6—O4	123.7 (4)
O4—Yb1—O3W	79.48 (11)	O3—C6—C5	118.9 (4)
O3^ii^—Yb1—O3W	140.62 (12)	O4—C6—C5	117.3 (4)
O1W—Yb1—O3W	138.30 (12)	C5—C7—C8	120.8 (4)
O2^i^—Yb1—O6	94.87 (11)	C5—C7—H7	119.6
O4—Yb1—O6	148.87 (11)	C8—C7—H7	119.6
O3^ii^—Yb1—O6	85.72 (11)	C2—C8—C7	120.8 (4)
O1W—Yb1—O6	133.53 (11)	C2—C8—H8	119.6
O3W—Yb1—O6	77.85 (11)	C7—C8—H8	119.6
O2^i^—Yb1—O2W	143.67 (12)	O5—C9—O6	119.7 (4)
O4—Yb1—O2W	74.76 (12)	O5—C9—C10	121.4 (4)
O3^ii^—Yb1—O2W	71.38 (12)	O6—C9—C10	118.8 (4)
O1W—Yb1—O2W	133.46 (12)	O5—C9—Yb1	62.0 (2)
O3W—Yb1—O2W	70.26 (12)	O6—C9—Yb1	57.76 (19)
O6—Yb1—O2W	77.66 (11)	C10—C9—Yb1	174.7 (3)
O2^i^—Yb1—O5	77.29 (11)	C12—C10—C11	118.9 (4)
O4—Yb1—O5	156.47 (11)	C12—C10—C9	121.2 (4)
O3^ii^—Yb1—O5	74.22 (11)	C11—C10—C9	119.9 (4)
O1W—Yb1—O5	79.44 (11)	C12^iii^—C11—C10	120.0 (4)
O3W—Yb1—O5	120.37 (11)	C12^iii^—C11—H11	120.0
O6—Yb1—O5	54.27 (9)	C10—C11—H11	120.0
O2W—Yb1—O5	121.90 (10)	C10—C12—C11^iii^	121.1 (4)
O2^i^—Yb1—C9	86.23 (11)	C10—C12—H12	119.5
O4—Yb1—C9	174.57 (12)	C11^iii^—C12—H12	119.5
O3^ii^—Yb1—C9	78.02 (11)	C1—O2—Yb1^i^	161.1 (3)
O1W—Yb1—C9	106.19 (13)	C6—O3—Yb1^ii^	162.9 (3)
O3W—Yb1—C9	100.18 (12)	C6—O4—Yb1	137.1 (3)
O6—Yb1—C9	27.39 (11)	C9—O5—Yb1	91.1 (2)
O2W—Yb1—C9	99.99 (11)	C9—O6—Yb1	94.8 (2)
O5—Yb1—C9	26.89 (11)	Yb1—O1W—H1W	121 (3)
O1—C1—O2	124.3 (4)	Yb1—O1W—H2W	132 (3)
O1—C1—C2	118.7 (4)	H1W—O1W—H2W	105 (5)
O2—C1—C2	116.9 (4)	Yb1—O2W—H3W	118 (4)
C8—C2—C3	118.3 (4)	Yb1—O2W—H4W	109 (4)
C8—C2—C1	121.2 (4)	H3W—O2W—H4W	105 (5)
C3—C2—C1	120.3 (4)	Yb1—O3W—H5W	119 (4)
C2—C3—C4	121.6 (5)	Yb1—O3W—H6W	119 (4)
C2—C3—H3	119.2	H5W—O3W—H6W	104 (4)
C4—C3—H3	119.2		

Symmetry codes: (i) −*x*+1, −*y*+1, −*z*+1; (ii) −*x*, −*y*+1, −*z*+2; (iii) −*x*+1, −*y*, −*z*+3.

### Hydrogen-bond geometry (Å, °)

**Table:**

*D*—H···*A*	*D*—H	H···*A*	*D*···*A*	*D*—H···*A*
O1W—H1W···O2W^ii^	0.81 (4)	2.35 (3)	3.123 (5)	158 (6)
O1W—H1W···O3	0.81 (4)	2.52 (4)	3.104 (5)	130 (4)
O1W—H2W···O5^iv^	0.82 (4)	1.91 (5)	2.714 (4)	170 (5)
O2W—H3W···O1^v^	0.82 (5)	1.97 (5)	2.771 (4)	167 (5)
O2W—H4W···O1^vi^	0.82 (3)	2.17 (3)	2.900 (5)	149 (5)
O3W—H5W···O6^vii^	0.82 (3)	2.08 (2)	2.846 (4)	156 (4)
O3W—H6W···O1^vi^	0.82 (4)	1.95 (4)	2.746 (4)	165 (5)

Symmetry codes: (ii) −*x*, −*y*+1, −*z*+2; (iv) −*x*+1, −*y*+1, −*z*+2; (v) −*x*, −*y*+1, −*z*+1; (vi) *x*, *y*−1, *z*+1; (vii) −*x*+1, −*y*, −*z*+2.
